# Pars Plana Vitrectomy in Inherited Retinal Diseases: A Comprehensive Review of the Literature

**DOI:** 10.3390/life13061241

**Published:** 2023-05-24

**Authors:** Claudio Iovino, Andrea Rosolia, Luciana Damiano, Clemente Maria Iodice, Valentina Di Iorio, Francesco Testa, Francesca Simonelli

**Affiliations:** Eye Clinic, Multidisciplinary Department of Medical, Surgical and Dental Sciences, University of Campania Luigi Vanvitelli, 80131 Naples, Italy

**Keywords:** best disease, choroideremia, familiar exudative vitreoretinopathy, pars plana vitrectomy, retinitis pigmentosa, Stargardt disease, X-linked retinoschisis

## Abstract

Inherited retinal diseases (IRDs) are a group of clinically and genetically heterogeneous disorders that may be complicated by several vitreoretinal conditions requiring a surgical approach. Pars plana vitrectomy (PPV) stands as a valuable treatment option in these cases, but its application in eyes with such severely impaired chorioretinal architectures remains controversial. Furthermore, the spreading of gene therapy and the increasing use of retinal prostheses will end up in a marked increase in demand for PPV surgery for IRD patients. The retinal degeneration that typically affects patients with hereditary retinal disorders may influence the execution of the surgery and the expected results. Considering the importance of PPV application in IRD-related complications, it is fundamental to try to understand from the literature what is adequate and safe in posterior eye segment surgery. Use of dyes, light toxicity, and risk of wounding scar development have always been themes that discourage the execution of vitreoretinal surgery in already impaired eyes. Therefore, this review aims to comprehensively summarize all PPV applications in different IRDs, highlighting the favorable results as well as the potential precautions to consider when performing vitreoretinal surgery in these eyes.

## 1. Introduction

Pars plana vitrectomy (PPV) is a commonly employed surgical technique that allows *ab-interno* access to the posterior segment of the eye [1]. The procedure consists of partial or extensive vitreous removal, with a successive execution of the required retinal surgery [1].

Inherited retinal diseases (IRDs) are a composite group of disorders characterized by retinal degeneration at different levels [2]. These conditions typically lead to progressive and significant vision impairment over time. Vitreoretinal abnormalities are particularly frequent in these eyes, but the indications and the way to execute the surgery itself may be different [3,4]. PPV was reported to be an effective alternative to manage clinical conditions such as vitreomacular interface disorders and retinal detachments (RD) [3,4].

Moreover, subretinal gene therapies and retinal prostheses are going to become quite common treatment options in hereditary retinal disorders, and a better understanding of the current and future PPV applications in these patients is warranted.

Therefore, this review aims to summarize and discuss the current literature on clinical applications of PPV in patients with IRDs.

## 2. Applications of Pars Plana Vitrectomy for Common Clinical Conditions in Inherited Retinal Diseases

All the principal clinical applications of PPV in hereditary retinal disorders are summarized in Table 1.

### 2.1. Pars Plana Vitrectomy for Epiretinal Membranes in Inherited Retinal Diseases

Epiretinal membrane (ERM) is a pathological pre-retinal film composed of a cellular inner layer of myofibroblast-like cells and an outer layer mainly formed by collagen fibrils randomly oriented, covering the inner limiting membrane (ILM) and leading to distortion of the normal retinal architecture [30]. The pathogenetic process that leads to an increased prevalence of ERM in IRDs is still not completely understood.

#### 2.1.1. Pars Plana Vitrectomy for Epiretinal Membrane in Retinitis Pigmentosa

It was speculated that the chronic inflammatory reaction characterizing the entire course of the disease might play a key role in ERM formation [31].

The prevalence of ERMs in patients with RP is estimated to range from 15.6% to 27.3% [32,33,34]. Triolo et al. observed that if ILM thickening is taken into account as well, the estimated frequency would even rise up to 94% [34]. Furthermore, Testa et al. noted an ERM prevalence within RP children of 8.6%, significantly higher when compared to the general pediatric population (about 1/21,000 children) [32].

Prior to surgery, an accurate assessment of the ellipsoid zone needs to be carefully carried out, as its integrity has been reported to significantly influence the postoperative functional outcome [5].

Ikeda et al. retrospectively evaluated the results of PPV with ILM peeling in 11 RP eyes with ERMs, demonstrating no relevant changes from baseline in terms of best-corrected visual acuity (BCVA) between treated and untreated eyes, whereas retinal anatomy strikingly improved with a marked central retinal thickness (CRT) reduction [5]. Conversely, Vingolo et al. enrolled eight RP patients with strong vitreomacular tractions (VMT), with or without coexistent ERMs [3]. Following phacoemulsification with an intraocular lens (IOL) implantation, a microincision vitrectomy surgery (MIVS) with 23-gauge trocars was performed with posterior hyaloid detachment, ERM and ILM peeling, and air-gas (SF6) exchange. In their report, the BCVA improvement was relevant, and spectral domain (SD)-OCT showed a considerable reduction in CRT [3].

With regard to ILM staining, Vingolo et al. reported no use of dye for ILM removal in RP patients, showing better results in BCVA compared to the study by Ikeda et al., who, instead, used brilliant blue G dye solution (0.25 mg/mL) [3].

Considering the similar mean preoperative BCVA, it can be speculated that one of the reasons for limited BCVA improvement in the work of Ikeda et al. may be related to the use of dyes. Worthy of note, Vingolo et al. performed phacoemulsification in all eyes, and Ikeda et al. did the same only in 45% of cases, and this may explain the different BCVA outcomes [3,5].

ILM peeling in RP patients with ERMs seems to be a proper choice, as MIVS without ILM peeling in RP patients was correlated to a BCVA reduction in a previous study [9]. Additionally, it was demonstrated that ILM peeling could also prevent the secondary development of macular holes (MHs) [35].

In summary, it was not completely understood whether performing PPV + ERM peeling in RP patients would ensure favorable visual outcomes, whereas an evident morphological improvement was described. Vitreoretinal surgery could represent an option in eyes with BCVA worsening due to ERM progression with still preserved photoreceptors.

#### 2.1.2. Pars Plana Vitrectomy for Epiretinal Membrane in Stargardt Disease

The data present in the literature regarding Stargardt disease (SGD) do not allow a clear perspective about the real advantages of PPV for ERM treatment. Indeed, ERMs in SGD have been known to undergo spontaneous separation in children [36]. Therefore, a conservative approach would be generally warranted, as surgical intervention appears to be described only in a few studies reporting contrasting postoperative anatomical and functional outcomes [6,7].

### 2.2. Pars Plana Vitrectomy for Macular Holes in Inherited Retinal Diseases

A MH is a defect of the central macula characterized by an interruption of both inner and outer retinal layers. Its development seems to arise from different possible mechanisms, such as dynamic tractional forces generated by vitreous and cellular shrinkage of residual hyaloid, causing elevation and retraction of retinal layers [37].

#### 2.2.1. Pars Plana Vitrectomy for Macular Holes in Retinitis Pigmentosa

Compared to an ERM, full-thickness macular hole (FTMH) is a relatively uncommon feature of RP patients, with a reported prevalence that ranges between 0.5−4.5% [32]. This finding, anyhow, is highly more frequent when compared to the general population [13,38], with a comparable female-to-male ratio [32].

The mechanism of MH development in RP is not fully elucidated, even though multifactorial pathogenesis has been proposed [39], which describes both a collapse of the blood-retinal barrier and dysfunction of Muller cells resulting in RPE pumping failure. This cascade of events may eventually provoke CME development, and the subsequent fusion of the cysts may facilitate the FTMH formation [40].

The indications about when and how to perform surgery of MHs in RP have never been discussed, and despite the remarkable clinical impact of MHs on the natural history of the disease, only small sample-sized works have been published [13].

This surgery has been reported to be more complicated than in the general population, as the retinal thinning caused by photoreceptors degeneration and the macular pigmentary changes may significantly impact the MH closure pattern [13]. Furthermore, the malfunction of RPE can easily lead to a failure of the pumping mechanism, causing fluid accumulation in the fovea and progressive enlargement of the hole, which may seriously affect both the surgical procedure and the postoperative outcomes [41].

Previous surgical treatments featuring classical ILM peeling and gas or silicon tamponade have shown controversial outcomes in terms of BCVA change [9,10,11,12], whereas flap-assisted techniques for RPs with macular holes have been recently described with favorable results [13]. More and more data are required to define which PPV technique of MH closure is the most performant and if any dyes for ILM staining or any gas tamponade may lead to better outcomes. In view of the results from the flap-assisted surgery performed by Lee et al., we might speculate that, in line with the general indications suggesting alternative techniques in case of challenging situations like large primary or recurrent macular holes, we may consider RP as an indication to approach the macular hole differently [13]. Larger-size cohort studies are needed to better evaluate these considerations.

#### 2.2.2. Pars Plana Vitrectomy for Macular Hole in Stargardt Disease

Vitreoretinal interface disorders in association with SGD have rarely been described. Moreover, considering the macular involvement that characterizes the natural history of the disease, the development of a macular hole does not have a great impact on central vision, which is indeed rather improbable to recover [42].

**Figure 1:**
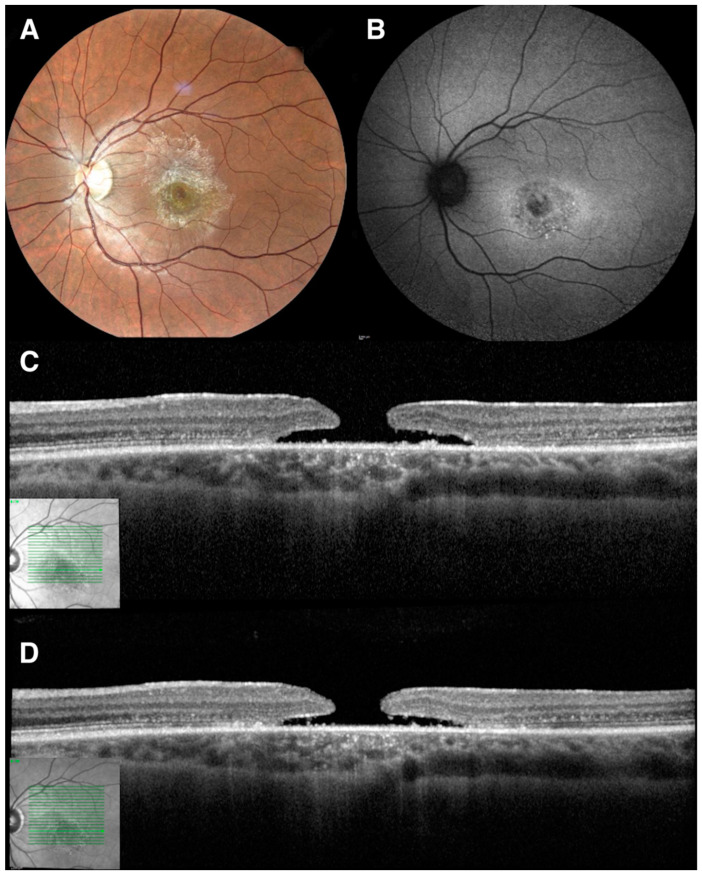
Multimodal imaging of a 40-year-old patient with genetically confirmed Stargardt disease (SGD) and full-thickness macular hole (FTMH). (**A**): True color 55° fundus picture showing the macular pre-retinal hyper-reflective band with a central macular hole. (**B**) Blue-light 55° autofluorescence displaying a central foveal hypo-autofluorescence surrounded by a hyper/iso-autofluorescent ring. (**C**) Spectral-domain optical coherence tomography (SD-OCT) scan showing a FTMH with a maximum diameter of 435 μm (**D**) Two-year follow-up tracked-SD-OCT displaying no progression of the FTMH with a maximum diameter of 432 μm.

The decision to perform a vitrectomy in these eyes is not easy, especially if considering the high risk of fibrosis due to the abnormal wound-healing response that may develop in SGD [43]. Rizzo et al. described in 2016 the first case in the literature of MH closure in Stargardt disease, achieved using 25-Gauge PPV with the removal of the ERM, peeling of the ILM, and fluid-air exchange with subsequent SF6 gas injection [14]. Postoperative outcomes resulted in significant improvement of both retinal morphology and BCVA, with no significant fibrosis observed during the 1-year follow-up. A modest postsurgical increase of the scotomas at microperimetry occurred in more than half of patients undergoing ILM peeling, and it was reported to not be related to surgery but rather to the natural course of the disease [14]. PPV could represent a therapeutic option to treat vitreoretinal interface abnormalities leading to hole closure in selected cases of SGD, but more reports are warranted to understand the most appropriate management of this condition [14]. Observation only could also be an option for FTMH in SGD patients, considering the relatively slow progression of the condition over time (Figure 1).

#### 2.2.3. Pars Plana Vitrectomy for Macular Hole in Choroideremia

MHs represent one of the possible features of the final stage of Choroideremia (CHM), with a prevalence estimated to be about 10% [15].

However, there are just a few details reported about the management of MHs in this condition [15]. The results described are not brilliant in terms of functional improvement, but they still show that it is possible to achieve anatomical MH closure in greatly damaged chorioretinal structures. The prospect of subretinal injections for gene therapy alerts us to the need to approach a MH in a retina affected by CHM [44]. Subretinal injection of the CHM gene requires continuous and uninterrupted retinal layers to allow proper delivery to the cells and to avoid its dispersion in the vitreous chamber; thus, a hole may prevent a successful treatment in these patients [15]. Moreover, a subretinal injection can itself be a possible cause of hole formation [44].

Two relevant observations about MH closure in CHM have been highlighted in the recent literature. First, choroidal and retinal atrophy progression following the surgical stress, including the ILM staining and peeling and the sustained light-pipe illumination, were not observed [15]. Secondly, gas resorption in CHM patients resulted in being significantly slower than in the general population, taking about twice as long to be dissipated. Therefore, the actual recommendation is to use short-duration gas tamponade as SF6 or even only air [15,45].

Furthermore, Ishikawa et al. reported a case of MH self-closure without surgery, which occurred following two months of treatment with topical betamethasone. Under this light, inflammation may play a pivotal role in MH development in late-stage CHM [46].

#### 2.2.4. Pars Plana Vitrectomy for Macular Hole in Best Disease

MHs have been described to develop in Best Disease [47].

The accumulation of vitelliform material induces a chronic separation of photoreceptors from the RPE layer, causing progressive macular atrophy [48]. The consequent retinal thinning is not only the cause of central vision loss that characterizes the natural history of the disease but also the presumed mechanism of MH development in these eyes [48].

SD-OCT analysis documented the advancement from the accumulation of hyperreflective material to foveal atrophy and then to FTMH development [48].

Previously, the rupture of vitelliform cysts has been considered another possible pathogenetic mechanism of hole development in these eyes, as secondary retinal changes due to the persistence of lipofuscin and induction of changes in the RPE with foveal dehiscence have been hypothesized [49,50].

Different surgical techniques have been described to realize hole closure. PPV with ILM peeling and gas tamponade with SF6 has been successful in a case described by Liu et al. [16], but it also required a second surgery with the use of heavy silicone oil [17]. Moreover, the inverted flap technique and SF6 gas tamponade allowed long-term good anatomical outcomes in two cases of MHs with associated RD [18].

### 2.3. Pars Plana Vitrectomy for Refractory Cystoid Macular Edema in Inherited Retinal Diseases

CME may complicate RP in about 10 to 50% of cases [32,33,34], and its management is basically pharmacological [51]. As in most non-inherited retinal diseases, the blood-retinal barrier breakdown has been described to lead to an uncontrolled fluid entry, as the dysfunctional RPE does not properly pump fluid into the choriocapillaris [52].

The possible impact of vitreous traction within this multifactorial pathogenesis may allow us to investigate the outcomes of a surgical approach [19]. To the best of our knowledge, the study of Garcia-Arumi et al. is the only one in the literature describing PPV to treat an RP-related CME [19]. They performed PPV with ILM peeling after indocyanine green (ICG) staining and gas tamponade in 12 eyes with a CME refractory to oral acetazolamide treatment, reporting significant post-operatory BCVA increase and CMT decrease [19]. Further studies are warranted to elucidate the proper indications and the optimal surgical timing of CME.

### 2.4. Pars Plana Vitrectomy for Retinal Detachment in Inherited Retinal Diseases

RD in IRDs is a feared complication, with an occurrence that is highly variable in different conditions [27,28,53]. Indeed, each of them shows characteristic features which can promote or hinder a detachment of the neuroretina.

#### 2.4.1. Pars Plana Vitrectomy for Retinal Detachment in Retinitis Pigmentosa

RD in patients with RP is a rare condition, with an estimated prevalence that varies from 0.7% to 1.3% [53].

Pigment migration that occurs in RP has been suggested to be a protective factor for the occurrence of RD, forming retinal adhesions to the RPE-Bruch’s membrane complex and thus creating a bond between the neurosensory retina and RPE which may protect against possible disjunctions [52]. RD in RP occurs relatively early compared to non-RP patients. The natural history of the disease determines a progressive narrowing of subretinal space due to photoreceptors death, with consequent scarring, pigment dispersion, and the development of firm adhesions in the advanced stages of the disease [54].

The initial symptoms of RD typically consist of the onset of flashes and peripheral acute visual field loss [55]. Nevertheless, considering that RP patients already have low or no peripheral vision, they are usually symptomatic only when macular involvement occurs [20]. Indeed, the delayed presentation, with a mean duration of symptoms of 12–14 months, explains the low percentage of “macula on” RDs upon diagnosis, which is strictly related to poor surgical visual outcomes [4,20,21].

Three different forms of RDs have been described in RP: rhegmatogenous, tractional, or exudative.

Rhegmatogenous RD is the most common in the general population, and its prevalence in RP has been reported to be 0.059%, with a median presentation at 32 years of age [4]. Full-thickness lesions in the neuroretina allow the entry of fluid secondary to vitreous synchysis in the subretinal space, developing retinal disjunction [21]. These retinal breaks usually originate within areas of peripheral retinal degeneration [21]. There is no concordance in the literature about the most common type of retinal break in RP [21].

Dave et al. reported that the prevalent category of retinal breaks was atrophic retinal holes, either in isolation or in association with lattice degeneration, while the scarcity of horseshoe tears may be consequent to the development of the characteristics adhesions which contrast the tractional forces of the posterior RD [4]. Moreover, they reported the inferotemporal quadrant as the most frequent site [4].

This prevalence of round holes was confirmed by Chan et al., who also reported lower rates of incidence of horseshoe tears and macular holes [21]. On the other hand, Rishi et al. found horseshoe tears as the most common breaks, with most of them located in the superotemporal area [20].

Tractional RDs are the result of anterior-posterior vitreous traction [56]. There are few descriptions in the literature about tractional detachments in IRDs. Hirahara et al. presented a case of RP with associated tractional RD, spontaneously resolved with posterior vitreous detachment after 8 months [57]. The incidence of PVD in RP is high, but the protective adherences between neuroretina and RPE may be helpful to block the detachment progression until the vitreous separation is complete [57]. Chan et al. reported three cases of ‘’macula on’’ tractional RD in RP patients undergoing surgery with an improvement in the final BCVA [21].

Finally, the exudative is the least common form of RD. It occurs when fluid collects in the subretinal space due to a blood-retinal barrier breakdown [58].

Chan et al. described 24 cases of exudative RDs in RP patients, but none of them underwent surgery. Some cases were managed with cryotherapy, but it was ineffective, and the final VA did not improve [21]. The most fitting threshold of cryotherapy or laser therapy in RP can be particularly challenging to set due to the poor tissue signs after treatment [21]. A *CRB1* gene mutation is the genetic feature most frequently associated with exudative RP, secondary to microaneurysm and telangiectatic abnormalities found in some of these patients, also defined as a Coats-like reaction [59]. The Coats-like serous RD is typically inferior and bilateral, with female preponderance [60,61].

PPV with internal drainage of subretinal fluid, cryotherapy, endolaser photocoagulation, and silicone oil injection was performed in a case of *CRB1* exudative RD [22]. Over the following few months after surgery, the reabsorption of subretinal fluid was particularly slow, but no new telangiectasias were reported. After the silicon oil remotion, the retina remained well attached [22].

There are several aspects to analyse when performing RD surgery in patients with RP. Considering that the vitreous is generally not detached in young IRD patients, the use of a stain such as triamcinolone acetonide may be useful to mark the vitreous and ensure a complete vitreous clearance from the retina [21]. It is further important to avoid overtreatment with laser titration until tissue necrosis, which can occur easily considering the lack of laser burns visualization in the RP retina [21].

Regarding the comparison between PPV and scleral buckling, no statistical differences were revealed in terms of functional outcomes and intraoperative complications rate [52].

In eyes with horseshoe tears and the absence of PVD, the scleral buckle may be recommended: indeed, during vitrectomy, it may be arduous to detach the vitreous over the areas of retinal breaks [21].

Moreover, during PPV, a posterior retinotomy with an external drain is recommended to evacuate the accumulated subretinal fluid, as the RPE pumping function may not be sufficient to avoid a long-standing persistence of liquid [21].

The primary reattachment rates after surgery due to RD in RP patients are reported to be similar to the general population, and the procedures allow favorable anatomical and functional outcomes, except for cases with a severe macular impairment due to the primary disease [21].

#### 2.4.2. Pars Plana Vitrectomy for Retinal Detachment in Familiar Exudative Vitreoretinopathy

Familiar exudative vitreoretinopathy (FEVR) is a congenital disease characterized by incomplete development of the retinal vascular system. RD occurs in 10% to 30% of cases, and it can present as either rhegmatogenous, tractional, or exudative [27].

There is an evident male predominance in rhegmatogenous RD, while no gender differences have been observed in the other subgroups [23,24]. The onset age for rhegmatogenous RD ranges from 9 to 30 years old, while tractional and exudative detachments are reported in younger patients (average age: 3.2 ± 4.6 years) [24,25]. Most patients are myopic, and considering the impact of the globe enlargement on RD development, this may explain why rhegmatogenous RD occurs later than exudative or tractional types [24].

The retinal breaks described in these patients include both atrophic holes and retinal tears with vitreous traction and are often located on the demarcation line between the vascular and the peripheral avascular retina in the temporal area, which is frequently a site of strong vitreoretinal adhesions, and, thus, a rather common site of iatrogenic, almond-shaped retinal breaks [23,27].

Scleral buckling (SB) represents the first surgical choice to treat rhegmatogenous RDs in FEVR, showing a higher primary reattachment rate compared to PPV, which should be performed alone or in combination with scleral buckling in difficult or refractory cases, such as posteriorly located breaks, bullous retrolental RDs or cases with severe vitreous traction or PVR [23,24,26,27].

Few studies describe a prophylactic approach to prevent rhegmatogenous RDs in FEVR achieved through retinal ablation, which is currently used to reduce late-phase detachment rates in retinopathy of prematurity (ROP), that is as well characterized by the presence of peripheral avascular retina and associated RD [62]. However, FEVR has specific features other than ROP, such as a solid vitreous adhesion around the vascular demarcation area. The treatment may be helpful, but the results indicate that close follow-up remains necessary because treatment with laser photocoagulation was described as not sufficiently effective [24,27].

The severe form of the disease features fibrovascular proliferation and consequent tractional RD; it usually occurs before birth, but the condition can continue after birth or reoccur, generally with a slower progression ability, in the first years of life [62].

Yamane et al. described the surgical management of this condition by performing SB as the first choice of treatment in most eyes, using a silicone sponge of 2–3 mm in diameter, removed or cut after managing the fibrovascular proliferation, in order to avoid affecting the eye growth [63]. Similarly to ROP, SB in these eyes reduces both the traction and the vascular activity and proliferation [64]. In some FEVR cases, as the vitreous traction cannot be completely excised, scleral-buckle surgery would aim to change the direction and reduce the vitreoretinal pulling, reducing the risk of RD occurrence and stabilizing the traction over time [63].

On the other hand, PPV was described to be performed in complex cases in which fibrovascular growth was located in the posterior area or was largely extended [63].

Photocoagulation was also reported to be used prior to surgery, with the rationale to help the intra-surgical stabilization of vascular proliferation [63].

Considering the possible contraction of fibrovascular proliferation after anti-vascular endothelial growth factor (VEGF) administration, their use was described to possibly make the vitreous inflexible and more arduous to budge or cut [63].

With regards to exudative RD in FEVR, cases described in the literature would support the idea that exudation originates from vascular hyperpermeability, showing good response to photocoagulation treatment in the early stages, as reported by Pendergast et al. [25].

#### 2.4.3. Pars Plana Vitrectomy for Retinal Detachment in X-Linked Retinoschisis

X-linked retinoschisis (XLRS) is the most common juvenile-onset IRD in males. It is due to a mutation of the retinoschisin (RS1) gene, which causes the loss of normal cellular adhesion and interactions, provoking progressive splitting of the retina secondary to the vitreous traction [28].

The complications responsible for vision loss typically occur in the first decade and mainly include progressive foveal schisis, RD, and vitreous hemorrhage [28]. XLRS is usually a slowly progressive disease, but when a continuous peripheral schisis threatens the macular area, it is unclear whether the best option might be close clinical observation or surgery [29].

It is evident that vitreous traction plays a fundamental role in the development and advancement of retinoschisis. Ikeda et al. observed that retinal schisis continues in most patients of the non-surgical group, and even laser photocoagulation was not effective in stopping this progression, with a final poor prognosis when compared to the group that underwent PPV. The surgically induced PVD was reported to have favorable effects in restoring the physiological retinal structure [65].

Furthermore, about 20% of patients with XLRS may develop RD, which is the most common complication requiring surgery in these eyes [28,29].

SB was reported to be a reasonable choice as it would help to avoid inducing PVD, which may easily provoke iatrogenic breaks [28]. It can be considered the primary option in case of peripheral breaks and in the absence of PVR [28].

If performing SB does not have the expected outcome, or when the breaks are posterior, or there are signs of PVR, PPV is generally performed, as Schulman et al. reported for the first time in 1985 [66]. Core vitrectomy and induction of PVD with fluid-air exchange is followed by introducing a tamponade, such as silicon oil or perfluoropropane gas (C3F8, 14 or 16%), both reporting favorable results [28,64]. PPV has also been combined to a cerclage using a 240 band, placed 2.5 mm posterior to the ora serrata, but more data are needed to clarify its possible superiority [28]. Moreover, inner retinal layer (IRL) retinectomy allows better visualization of outer retinal layer breaks and ensures total vitreous detachment, helping to better fill up the breaks and dispel the traction [67]. However, the surgical outcomes appeared similar when comparing eyes that underwent IRL retinectomy to eyes which did not [28].

PVR is a historically feared postoperative complication of vitrectomy in XLRS [68], but more recent work with a longer follow-up stated that PPV outcomes in these patients have not considerably been influenced by PVR development, underlying how a careful, complete vitreous removal contributed to reducing this rate [29]. ILM peeling may be particularly risky for iatrogenic macular holes and retinal breaks development in XLRS; thus, a complete peeling is not considered mandatory, but instead, a partial removal around the retinoschisis area is suggested to reduce the need for a second surgery [29].

In summary, the surgical procedures applied differ in the various reports, but the outcomes achieved are comparable, showing vitreoretinal surgery to be superior to clinical observation [28,29].

## 3. Specific Applications of Pars Plana Vitrectomy in Inherited Retinal Diseases

### 3.1. Pars Plana Vitrectomy with Subretinal Injection in Inherited Retinal Diseases

Subretinal injection stands as a widely used method that allows targeted drug delivery, specifically adopted in gene therapy for IRDs [69]. Inoculating within the subretinal space allows substances to be delivered right in contact with photoreceptors and RPE cells [68]. Differently from an intravitreal injection, this procedure generates higher local concentrations of molecules that significantly improve the percentage of cells transduced and, thus, the overall therapeutic effect [69].

The first commercially available gene therapy was Voretigene Neparvovec-rzyl (Luxturna), an Adeno Associated Virus (AAV-2) vector carrying a copy of the *RPE65* gene, which was approved by the FDA in December 2017 and designed to treat retinal dystrophies associated to mutations in the *RPE65* gene, such as Leber Congenital Amaurosis type 2 (LCA2) and a subgroup of autosomal recessive RP [70].

The procedure requires advanced surgical skills and consists of a 3-port PPV with a retinotomy performed under general anesthesia with a 41-gauge cannula between the fovea and the temporal vascular arcades. The subretinal fluid injection determines a small, localized RD, after which a 50% fluid-air exchange is realized. The patient is kept supine for 24 h to allow a successful retinal reattachment [71]. To reduce inflammation deriving from surgery or unexpected immune responses, 1 mg/kg/day of Prednisone is given from 3 days prior to surgery and up to 7 days later, followed by a further 0.5 mg/kg/day for the following 10 days [71].

**Figure 2:**
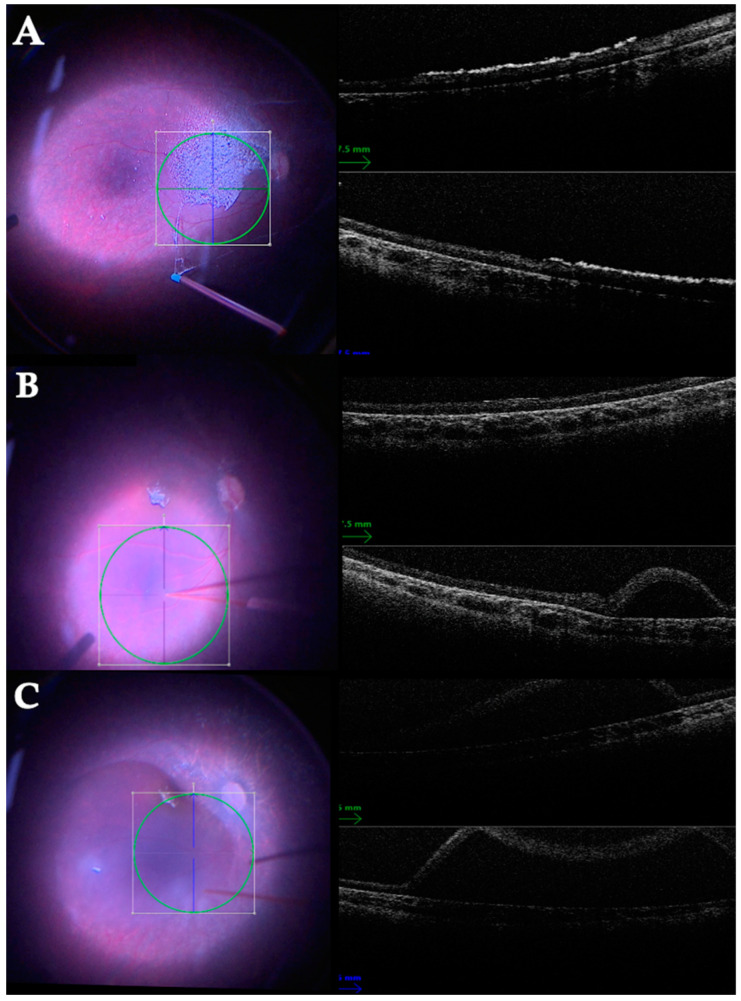
Representative case of an 11-year-old child with an *RPE65* mutation receiving a subretinal injection of Voretigene Neparvovec-rzyl (Luxturna) vector carrying a copy of the *RPE65* gene. (**A**) Following core vitreous removal, the strongly adherent posterior hyaloid membrane is visualized with triamcinolone acetonide (TA) injection and detached with a Charles cannula. Intraoperative optical coherence tomography (OCT) shows hyperreflective TA crystals on the retinal surface. (**B**) A 41-gauge cannula enters the retina along the superior temporal vascular arcade. The fluid injection underneath the retina determines a small, localized RD, which can be efficiently visualized with intraoperative OCT. (**C**) The localized RD enlarges, reaching the macula. The subretinal fluid is progressively injected under the guidance of the intraoperative OCT until the desired amount of fluid is delivered.

The use of optical coherence tomography (OCT) integrated with the operating microscope significantly helps to perform subretinal injections [72]. A representative case of a subretinal injection of Voretigene Neparvovec-rzyl is shown in Figure 2.

The functional outcomes after surgery showed an advantageous benefit-to-risk profile, with improvement in navigational ability and light sensitivity [73,74].

The safety profile is good, and most of the complications are mainly related to the procedure itself, except for a small portion of patients who can develop progressive perifoveal chorioretinal atrophy [73,75].

An optimized protocol for subretinal drug delivery was recently published by Reichel et al. They reported that a simple lavage of intravitreal fluid could effectively reduce the vector concentration. Considering that the intravitreal space is not an immune-privileged locus, exactly like the subretinal, this should be considered when executing subretinal gene therapy [76].

However, further studies are still necessary to determine which features may increase the success rate of this procedure. In fact, Gardiner et al. reported that retinal degeneration might carry on even after treatment [77].

RPE65 is not the only target gene considered to perform gene therapy in IRDs. Indeed, several clinical trials are in progress to target major genes, such as CHM in Choroideremia, MERTK in RP, CNGA3, and CNGB3 in Achromatopsia, and many others [78].

Another application for subretinal injections in IRDs is cell therapy. In fact, both photoreceptors and RPE cells may be theoretically replaced using stem cells [79]. These therapies are still not approved for clinical use, as different phase I and II trials are still investigating their safety [79]. Various cell types have been used as reliable sources of therapeutic cells, among which human embryonic stem cells (hESCs), pluripotent stem cells (iPSCs), and human umbilical tissue-derived cells (hUTCs) have been considered [80].

Cell therapy is usually performed through standard subretinal injections when a bolus of cells is injected, whereas a more complex surgery is required when delivering a monolayer patch of RPE cells, which should reduce the rate of transplant failure caused by dysfunction in the formation of cellular junctions [79].

Kashani et al. transplanted a patch of RPE cells in AMD patients [81]. They used a 41-gauge subretinal infusion cannula to elevate a bleb next to the atrophic area, and then a curved cannula was used to separate the retina overlying the atrophy. The retinotomy was enlarged to about 1 mm, after which the patch was loaded [81]. Perfluorocarbon heavy liquid (PFC) flattened the retina, and the retinotomy was closed with laser retinopexy. An air-fluid exchange was performed, PFC was removed, and then expansile gas or silicone oil was injected [81].

This technique may represent a future alternative option for cell therapy in IRDs.

### 3.2. Pars Plana Vitrectomy for Retinal Prosthesis in Inherited Retinal Diseases

A retinal prosthesis is a surgically implantable device that electrically stimulates the pathway that generates vision [82]. The system allows replacing the phototransduction cascade, which transmits the electrical signals up to the visual cortex. Most of these devices are epiretinal, adjoining the retinal ganglion cell layer, while others are subretinal, requiring intact middle and inner retinal layers to work efficiently [82].

The Argus II retinal prosthesis system was the first device to obtain authorization for clinical use [83]. Patients with RP over 25 years of age with bare-light or no-light perception are candidates for treatment [83]. The device is made up of external components, namely a pair of goggles with an integrated camera, a video processing unit, and an external coil able to transmit data and power to the internal components. An internal coil converts radio waves to electric signals, and through an inbuilt application-specific-integrated circuit (ASIC), these electrical pulses arrive in a 60-channel microelectrode epiretinal array [83].

The surgical implantation of the Argus^®^ II Retinal Prosthesis starts with a 360° peritomy to isolate all four recti muscles. The internal coil is positioned under the lateral rectus muscle, and the remnant silicone band is placed under the other rectus muscles and sutured onto the sclera in the superior-nasal quadrant at a pre-determined distance from the limbus, which is usually about 5mm. A standard 3-port PPV is performed, and the posterior hyaloid, along with any potential epiretinal membrane, is removed. Next, a 5.2-mm wide sclerotomy in the superior-temporal quadrant, at about 3 mm from the limbus, allows the introduction of the microelectrodes in the macular region. When the array position is adequate, a titanium retinal tack is placed at its heel to secure it. The sclerotomy is sutured closed after a 2 to 4 h surgery [83].

Argus II in RP patients was reported to help achieve better motion tasks when compared to the original vision with a good safety profile [84].

On the other hand, the retinal implant alpha-AMS represents a subretinal option to treat degenerative outer retinal diseases such as RP and Cone-Rod Dystrophy. The implantation needs from 6 to 8 h. It starts with a first arcuate retro-auricular incision and a secondary horizontal, which crosses the first; thus, a recess in the mastoid bone is made to host a casing containing the induction coil. Next, a skin and periosteal incision over the superolateral orbital rim and a subcutaneous tunnel between the two incisions were made, allowing passage for both the retinal implant and a cable from behind the ear to the orbit. A complete peritomy is performed at the limbus, and a subconjunctival tunnel to the orbital rim is made [85]. The intraocular procedure begins with a 20-gauge PPV followed by the creation of a scleral flap located 9 mm posterior to the limbus in the superior-temporal quadrant. A macular subretinal bleb is made by injecting a balanced salt solution with an extendible 41-gauge cannula, and a guiding foil is inserted through the sclera and choroid to a subretinal position, allowing positioning of the retina implant. After the remotion of the guiding tool, the cable is sutured to the sclera with an integrated silicone mesh, which is finally covered with a scleral patch graft. A silicon oil tamponade is injected at the end of the procedure [85].

Further clinical trials are warranted to test other types of prostheses to better evaluate their outcomes and cost-effectiveness.

## 4. Toxicity and Procedure-Related Risks of Pars Plana Vitrectomy in Inherited Retinal Diseases

The use of dyes, the light toxicity, and the risk of scar development have always discouraged the execution of vitreoretinal surgery in eyes with IRDs [8,86,87,88,89,90]. Considering the importance of PPV in treating IRD-related complications or in the delivery of subretinal drugs and in the placement of retinal prostheses, it is fundamental to discuss and summarize the safety reports from the current literature [8,86,87,88,89,90].

Phototoxicity is a well-known phenomenon secondary to retinal exposition to high-intensity light [86]. In modern vitreoretinal surgery, the development of stronger xenon light sources would increase the risk of toxicity due to their short wavelength, but coupling these devices with chandeliers allowed to extend the working distance, with a remarkable damage reduction [87]. The introduction of light-emitting diodes (LED) coupled with small gauge instruments (25 G–27 G) provided further limiting of the total amount of dissipated light. Moreover, the use of spectral filters has also played a role in reducing the phototoxicity phenomenon [87].

Several medical devices have been used to conduct vitreoretinal surgery in IRDs. Ultra-short-acting dyes have been adopted to better visualize ILMs and ERMs. The toxicity of ICG is widely described in the general population [88]. ERM surgery in RP has been performed using ICG, and various cases developed retinal degeneration [11,89]. The use of ICG should be avoided in these eyes if possible and absolutely forbidden if performing MHs or RD surgery, in which scenarios the ICG can directly enter into contact with the RPE [90]. On the other hand, Brilliant Blue G in the ERM surgery of RP has been reported to be useful in staining the ILM and considered to not cause intraoperative damage nor affect the surgical outcomes [5]. However, Vingolo et al. assessed that ILM peeling in RP might also be conducted, avoiding any staining thanks to its characteristic thickness, thus completely preventing any potential cromotoxicity [3].

With regard to the use of tamponades in PPV, it remains fundamental [8]. However, considering the damaged chorioretinal structure of some IRDs and the limited capacity of clearance of these materials, their abnormally prolonged persistence may be harmful [15]. Tamponade gases have been used: SF6 exhibited good results in CHM, where the reabsorption issue, as in RP, was described to be particularly relevant [8,15].

The risk of scar development secondary to vitreoretinal surgery is another current concern in patients with IRDs [90]. It has been suggested that an impaired retinal architecture, as in IRD patients, would make them more prone to post-surgical fibrosis [90]. Moreover, a recent work described the development and evolution of perifoveal chorioretinal atrophy following Subretinal Voretigene Neparvovecrzyl for RPE65-Mediated Leber Congenital Amaurosis [75].

Nevertheless, more studies are required to better understand if a standardized, optimized surgical procedure may avoid the risk of fibrotic and/or atrophic responses after vitreoretinal procedures.

## 5. Conclusions

Performing PPV in IRDs is certainly different if compared to the general population.

Various features characterizing the different hereditary retinal conditions can influence the surgical procedure as well as the functional outcomes. The potential toxicity related to the use of dyes and light sources and the risk of wounding scars development should be carefully considered. Nevertheless, in selected cases, PPV could be considered as an alternative to manage IRD-related complications, including vitreomacular disorders and RD. Moreover, vitreoretinal surgery also allows the treatment of IRDs through the implant of a retinal prosthesis and the delivery of gene therapies directly into the subretinal space.

In conclusion, taking into account all the aforementioned considerations when performing PPV in IRD patients may help in achieving good anatomical and functional outcomes even in eyes with an already impaired chorioretinal structure.

## Figures and Tables

**Table 1:** Clinical applications of pars plana vitrectomy in inherited retinal diseases.

Retinal Condition	IRD	Authors	Year	Number of Eyes	Surgical Method	BCVA Change
Epiretinal Membrane	RP	Vingolo et al. [3]	2014	8	PPV, ILM peeling with no dyes, and SF6 injection	Improved
		Ikeda et al. [5]	2015	11	PPV with triamcinolone acetonide, ILM peeling with brilliant blue G dye	Unchanged
	SGD	Potter et al. [6]	1995	1	PPV with ERM removal	Improved
		Bhende et al. [7]	2015	2	PPV with ERM removal	Improved
Macular Hole	RP	Vingolo et al. [8]	2015	3	PPV, ILM peeling, and silicon injection	Improved
		Hagiwara et al. [9]	2011	2	PPV, ILM peeling, SF6 injection	1 eye improved, 1 eye worsened
		Jin et al. [10]	2008	3	PPV, ILM peeling, C3F8 injection	3 eyes improved
		Yan et al. [11]	2018	4	PPV, ILM, SF6 or C3F8 or silicon injection	2 eyes improved, 1eye unchanged, 1 eye worsened
		Garcia-Fernandez et al. [12]	2013	1	PPV, ILM peeling SF6 injection	Unchanged
		Lee et al. [13]	2021	1	PPV, ILM peeling, and C3F8 injection	Improved
				1	PPV, ILM flap technique, and C3F8 injection	Improved
	SGD	Rizzo et al. [14]	2017	1	PPV, ILM peeling SF6 injection	Unchanged
	CHM	Zinkernagel et al. [15]	2013	3	PPV, ILM peeling, SF6 or C3F8 injection	Unchanged
	BEST	Liu et al. [16]	2016	2	PPV, ILM peeling, and SF6 injection	Unchanged
		De Souza et al. [17]	2012	1	PPV, ILM, and silicon injection	Improved
		Tewari et al. [18]	2018	2	PPV, ILM flap technique, and SF6 injection	Improved
Cystoid Macular Edema	RP	Garcia-Arumi et al. [19]	2003	12	PPV, ILM peeling with ICG and gas tamponade	Improved
Retinal Detachment	RP	Dave et al. [4]	2016	6	PPV with endolaser and silicon injection	Unchanged
		Rishi et al. [20]	2017	10	PPV with gas or silicon injection	Improved
		Chan et al. [21]	2020	12	PPV, retinopexy, and gas or silicon injection	Unchanged
		Lee et al. [22]	2004	1	PPV, cryotherapy, endolaser, and silicon injection	Unchanged
	FEVR	Ikeda et al. [23]	1999	25	PPV, SB, lensectomy, gas or silicon injection	Improved partially
		Chen et al. [24]	2012	6	PPV, SB, lensectomy, gas or silicon injection	Improved
		Pendergast et al. [25]	1998	26	PPV	Improved partially
				1	PPV and SB	Improved partially
		Sen et al. [26]	2020	30	PPV, SB, gas, or silicon injection	Improved partially
		Katagiri et al. [27]	2017	6	PPV, gas, or silicon injection	Improved partially
				3	PPV and SB, gas or silicon injection	Improved partially
	XLRS	Sen et al. [28]	2018	22	PPV, C3F8, or silicon injection	Improved
		Yu et al. [29]	2012	17	PPV, ILM peeling, photocoagulation, and gas injection	Improved

BCVA: best corrected visual acuity; CHM: choroideremia; ERM: epiretinal membrane; FEVR: familiar exudative vitreoretinopathy; ILM: internal limiting membrane; IRD: inherited retinal disease; PPV: pars plana vitrectomy; RP: retinitis pigmentosa; SB: scleral buckling; SGD: Stargardt disease; XLRS: X-linked retinoschisis.

## Data Availability

Data sharing is not applicable to this article as no datasets were generated or analyzed in the current article.
